# Supplementary material for: The genome sequence of the Spruce-seed moth, Cydia strobilella (Linnaeus, 1758)
Source: Wellcome Open Res. 2024 Apr 2;9:177. [Version 1] doi: 10.12688/wellcomeopenres.21214.1 (PMC11422755; doi:10.12688/wellcomeopenres.21214.1)
Supplement: Peer review history

# Reviewer report: Reviewer response for version 1

**DOI:** 10.21956/wellcomeopenres.23461.r98386

**Published:** 2024-09-24

**Authors:** Gandhi Gracy, Selva Babu Selvamani

**Affiliations:** 1 Division of Genomic Resources, ICAR - National Bureau of Agricultural Insect Resources, Bengaluru, Karnataka, India

In the present study, the authors have presented a high-definition genome reference of *Cydia strobilella*.

 This will be a source for the gold-standard reference genome of this species and can be used in future studies that are going to be conducted on this organism. The BUSCO score of 98.2% explains the completeness of the presented genome. The provided graphs are understandable, particularly BlobToolkit results and Hi-C scaffolding. 

 In the case of data access and retrieval, the data is easily accessible from the provided databases. 

 However, addressing some review points will benefit the manuscript and be helpful for the readers.

1. The background and significance of sequencing the organism are not mentioned in detail, providing the information on the pest status and economic importance will improve the manuscript
2. Why was the male genome sequenced? What about female moths?
3. The mitochondrial genome size was mentioned as 16.78 KB (0.01678 Mb) but in the table the same as mentioned as 0.02 Mb (reason for round-off?)

Are sufficient details of methods and materials provided to allow replication by others?

Yes

Is the rationale for creating the dataset(s) clearly described?

Partly

Are the datasets clearly presented in a useable and accessible format?

Yes

Are the protocols appropriate and is the work technically sound?

Yes

Reviewer Expertise:

Molecular Entomology, Insect Genomics and Bioinformatics

We confirm that we have read this submission and believe that we have an appropriate level of expertise to confirm that it is of an acceptable scientific standard.

# Reviewer report: Reviewer response for version 1

**DOI:** 10.21956/wellcomeopenres.23461.r98387

**Published:** 2024-09-21

**Authors:** Md-Mafizur Rahman

**Affiliations:** 1 Kangwon National University, Chuncheon-si, Gangwon-do, South Korea

**Title: The genome sequence of the Spruce-seed moth, *Cydia strobilella *(Linnaeus, 1758)**

 The research study is well-executed and holds potential for acceptance, but I have some concerns regarding the following points:

 The authors should ensure that the **Methods** section is detailed enough for replication:

- **Comment 1**: The background section could benefit from a more detailed explanation of why the genome sequencing of *Cydia strobilella* is significant within the Darwin Tree of Life Project. Clarify the broader biological and ecological importance of this species in the introduction.
- **Comment 2**: While the Hi-C data and manual curation are mentioned, more detail on the exact **assembly tools** and parameters used for the genome assembly would improve replicability.
- **Comment 3**: While 36-fold coverage may be adequate for small genomes or non-repetitive regions, complex regions (e.g., repetitive sequences or GC-rich areas) could benefit from higher coverage to reduce gaps and improve the overall assembly. Additionally, higher coverage might help in resolving heterozygosity and structural variants, which are especially important for understanding evolutionary dynamics in species like *Cydia strobilella*.

Are sufficient details of methods and materials provided to allow replication by others?

Yes

Is the rationale for creating the dataset(s) clearly described?

Yes

Are the datasets clearly presented in a useable and accessible format?

Yes

Are the protocols appropriate and is the work technically sound?

Yes

Reviewer Expertise:

Molecular biology ( microbial and insect genomics)

I confirm that I have read this submission and believe that I have an appropriate level of expertise to confirm that it is of an acceptable scientific standard.
